# Fast and Efficient Genome Editing of Human FOXP3^+^ Regulatory T Cells

**DOI:** 10.3389/fimmu.2021.655122

**Published:** 2021-08-02

**Authors:** Lauren Van Zeebroeck, Rebeca Arroyo Hornero, Beatriz F. Côrte-Real, Ibrahim Hamad, Torsten B. Meissner, Markus Kleinewietfeld

**Affiliations:** ^1^Vlaams Instituut voor Biotechnologie (VIB) Laboratory of Translational Immunomodulation, Vlaams Instituut voor Biotechnologie (VIB) Center for Inflammation Research (IRC), Hasselt University, Diepenbeek, Belgium; ^2^Department of Immunology, Biomedical Research Institute, Hasselt University, Diepenbeek, Belgium; ^3^Department of Surgery, Beth Israel Deaconess Medical Center, Harvard Medical School, Boston, MA, United States

**Keywords:** regulatory T cell, CD4, IL6R, CRISPR, human, genome editing, COVID-19, autoimmunity

## Abstract

FOXP3^+^ regulatory T cells (Tregs) are central for maintaining peripheral tolerance and immune homeostasis. Because of their immunosuppressive characteristics, Tregs are a potential therapeutic target in various diseases such as autoimmunity, transplantation and infectious diseases like COVID-19. Numerous studies are currently exploring the potential of adoptive Treg therapy in different disease settings and novel genome editing techniques like CRISPR/Cas will likely widen possibilities to strengthen its efficacy. However, robust and expeditious protocols for genome editing of human Tregs are limited. Here, we describe a rapid and effective protocol for reaching high genome editing efficiencies in human Tregs without compromising cell integrity, suitable for potential therapeutic applications. By deletion of *IL2RA* encoding for IL-2 receptor α-chain (CD25) in Tregs, we demonstrated the applicability of the method for downstream functional assays and highlighted the importance for CD25 for *in vitro* suppressive function of human Tregs. Moreover, deletion of *IL6RA* (CD126) in human Tregs elicits cytokine unresponsiveness and thus may prevent IL-6-mediated instability of Tregs, making it an attractive target to potentially boost functionality in settings of adoptive Treg therapies to contain overreaching inflammation or autoimmunity. Thus, our rapid and efficient protocol for genome editing in human Tregs may advance possibilities for Treg-based cellular therapies.

## Introduction

CD4^+^FOXP3^+^ regulatory T cells (Tregs) are essential for maintaining immune homeostasis and peripheral tolerance (1, 2). They are characterized by the expression of the transcription factor FOXP3 and CD25 (1, 2). Tregs can suppress several cell subtypes, such as T cells, B cells, natural killer (NK) cells and antigen presenting cells (APCs). Moreover, they have numerous modes of action: e.g. secretion of immunosuppressive cytokines [IL-10 (3), TGF-β (4), IL-35 (5)], induction of cell death by perforin and granzyme (6), IL-2 deprivation (7), CTLA-4-regulated downregulation of CD80/86 on APCs (8) and depletion of extracellular ATP and adenosine generation by CD39 (9, 10). Due to their immunosuppressive characteristics, numerous studies are currently exploring the potential of Treg cellular therapy for the induction of tolerance to autoantigens and alloantigens in the context of autoimmunity and transplant rejection (1, 2, 11–13). In addition, novel genome editing techniques like CRISPR (clustered, regularly interspaced, short palindromic repeats)/Cas9 (CRISPR-associated protein 9) holds great promise in enhancing the efficacity of Treg cell therapy. Tregs could be genetically engineered to target molecules that regulate Treg functionality disabling pathways that lead to instability or forcing the expression of molecules that enhance their function (14). Several studies have shown that environmental cues may control Treg stability (15, 16), with the effects of IL-6 regulating Treg balance and function being particularly well-studied (17–21). Elevated IL-6 levels are found in systemic lupus erythematosus (SLE), relapsing-remitting multiple sclerosis (MS), rheumatoid arthritis (RA) and inflammatory bowel disease (IBD) patients [reviewed in (22)]. Also, critically ill COVID-19 patients exhibit a so-called “cytokine storm”, with acute increase in pro-inflammatory cytokines such as IL-6, leading to immune cell infiltration in the lungs (23). IL-6 signaling blockade by tocilizumab, a monoclonal Ab (mAb) against IL-6 receptor (CD126), is an approved treatment for certain autoimmune disorders such as RA, and is also being tested for the treatment of COVID-19, with current data showing reduction in the rate of mechanical ventilation or death in severely ill patients, when given at the right time point (24–28). Interestingly, Tregs isolated from autoimmune patients showed acquisition of pro-inflammatory cytokines and impaired suppressive function (1, 2) and current evidence suggests alterations in Tregs in severely ill COVID-19 patients compared to patients with a mild form of the disease (29), which could potentially contribute to excessive immune responses. Therefore, adoptive Treg therapy using genetically-engineered Tregs with enhanced stability in the presence of pro-inflammatory IL-6 environments could be a promising treatment in the context of autoimmune and infectious diseases.

Pioneering clinical trials using CRISPR/Cas9 in T cells have started. For example, *ex vivo* edited autologous PD-1 knock out (KO) T cells were used for treating advanced esophageal cancer (NCT03081715). Another example, in the field of CAR T cells, is the CRISPR-mediated deletion of PD-1 and replacement of the endogenous T cell receptor (TCR) by a cancer-specific TCR transgene for the treatment of advanced refractory myeloma and metastatic sarcoma (NCT03399448). Results of the latter clinical trial were recently published and showed that engineered T cells persisted up to nine months *in vivo*, demonstrating the feasibility of multiplex *ex vivo* CRISPR-mediated targeting for cancer therapy (30). Next-generation CRISPR techniques that avoid DNA double-strand breaks could minimize potential off-target effects and are likely to further improve its value for therapeutic applications (31). There are different methods to deliver CRISPR components to cells, both viral as well as non-viral methods. A non-integrative viral system is desirable for clinical application, with adeno-associated viral (AAV) vectors being a leading candidate for the delivery of CRISPR components. However, recent data have shown that persisting Cas9 expression could elicit an immunological response that may lead to the elimination of gene-edited cells (32). Non-viral approaches consisting of transient Cas9 expression include Cas9 and gRNA delivered as episomal plasmid DNA, mRNA or as recombinant Cas9 protein complexed together with the gRNA, also called ribonucleoprotein complexes (RNPs) (33). RNPs are currently the most attractive approach for the delivery of CRISPR components and several studies have demonstrated higher KO efficiencies by nucleofection of RNPs in activated human primary T cells (34). In contrast to total CD4^+^ primary T cells, genome editing in Tregs is less established since Tregs are anergic, more difficult to maintain in culture and only represent a minor fraction of cells in peripheral blood and other tissues (1, 2). Although previous studies have demonstrated that gene editing in Tregs is feasible, more efficient and rapid protocols are warranted.

Here, we described a rapid and effective protocol for gene KO in human Tregs using RNPs, suitable for potential therapeutic applications. We demonstrated high KO efficiencies without compromising FOXP3 expression or functionality. We validated that gene-edited Tregs can be efficiently used for downstream applications in functional assays, showing that *IL2RA*-KO Tregs have defective STAT5 signaling and suppressive function. Finally, we applied this protocol to investigate the role of the α-subunit of the IL-6 receptor (CD126) on Tregs. Our data showed that human *IL6RA*-KO Tregs do not activate STAT3 signaling in the presence of IL-6, suggesting that they may be resistant to IL-6-mediated instability, highlighting a potential therapeutic intervention to enhance Treg function in pro-inflammatory environments.

## Materials and Methods

### Treg Isolation

Peripheral blood mononuclear cells (PBMCs) were isolated by Ficoll (GE17-1440-03, Sigma-Aldrich) gradient centrifugation from buffy coats from healthy donors in compliance with institutional review board protocols (CME2019/042 and CME2016/629). CD4^+^ T cells were isolated using RosetteSep™ Human CD4^+^ T Cell Enrichment Cocktail (15062, Stemcell Technologies) according to manufacturer’s protocol. CD25^+^ T cells were isolated from PBMCs or CD4^+^ T cells using the Human CD25 MicroBeads II kit (130-097-044, Miltenyi Biotecs) according to manufacturer’s protocol and subsequently Tregs were sorted from these cells as propidium iodide (PI)^-^CD4^+^CD25^+^CD127^-^ on a FACS Aria II (BD Biosciences).

### Treg Stimulation

After isolation, Tregs were cultured for six days in 24-well plates at 250.000 cells/well in 1 mL X-vivo (BE02-060F, Lonza) + 5% heat-inactivated fetal bovine serum (FBS) (S1400, Biowest) with 10 µg/mL plate-bound anti-CD3 (555329, BD Biosciences), 1 µg/mL soluble anti-CD28 (555725, BD Biosciences) and 300 U/mL IL-2 (11147528001, Sigma-Aldrich) or 1500 IU/mL Proleukin^®^ (Novartis). For short term expansion experiments, Tregs were cultured for 24 hours in above-mentioned conditions and underwent subsequent nucleofection without prior re-plating to 6-well plates.

### TSDR DNA Methylation Analysis

Methylation at the Treg-Specific Demethylated Region (TSDR) was studied in 7 day-*in vitro* expanded CD4^+^CD25^-^CD127^+^ T conventional cells (Tconv) and CD4^+^CD25^+^CD127^-^ Tregs from matched donors. Genomic DNA was extracted from frozen samples using QIAamp DNA blood mini kit (51104, Qiagen) according to manufacturer’s protocol. Methylation analysis was performed by EpigenDx (Hopkinton, USA) by pyrosequencing of bisulfite-converted DNA. Nine representative CpG residues in the TSDR were analyzed using ADS783-FS2 assay for human FOXP3.

### Treg Nucleofection

24 hours prior to nucleofection, cells were cultured in 6-well plates at a density of 250.000 cells/mL in 2 mL X-vivo + 5% FBS and 100 U/mL IL-2 (11147528001, Sigma-Aldrich) or 500 IU/mL Proleukin^®^ (Novartis). For transfection, cells were collected, centrifuged at 90g for 10 minutes at room temperature, and 1 million Tregs were resuspended in 20 µl P3 Primary Cell 4D-Nucleofector X Kit S (V4XP-3032, Lonza). In PCR tubes, 20 pmol Cas9 nuclease (9212-0.25MG, Aldevron) was mixed with 100 pmol sgRNA (Synthego, **Table S1**) and incubated at 37°C for a minimum of 10 minutes before adding to the cells. For multiplexing, RNP complexes for each sgRNA were generated separately and equal amounts of each sgRNA was added. The cell/RNP mixture was transferred to Nucleofection cuvette strips (4D-Nucleofector X Kit S, Lonza) and cells were electroporated using the 4D-Nucleofector Core Unit (AAF-1002B, Lonza) and X Unit (AAF-1002X, Lonza) with program EO115. After transfection, 80 µl medium at room temperature (X-vivo + 5% FBS + 100 U/mL IL-2 or 500 IU/mL Proleukin^®^) was added to the wells of the cuvette strip. Cells were collected and plated in 1 mL pre-warmed medium in 24-well plates and incubated at 37°C until read-out. For re-stimulation, cells were activated 2 hours to 4 days after nucleofection with anti-CD3 plate bound mAb (1 – 10 µg/mL) and 1 µg/mL soluble anti-CD28 (555725, BD Biosciences) in the presence of IL-2.

### gRNA Design

gRNAs targeting *B2M*, *CD4* and *IL2RA* were described before (**Table S1**). gRNAs targeting *IL6RA* were designed using Nucleotide (NCBI) and CRISPOR (http://crispor.tefor.net/) and tested for their *in vitro* targeting efficiency.

### gRNA Activity Testing in HEK Cells

Ten gRNAs targeting *IL6RA* were designed and tested in HEK293T cells for their *in vitro* effectiveness of creating indels as described before (35). Briefly, HEK293T cells were transfected using jetOptimus buffer (Polyplus, #117-07) with 300 ng Cas9 plasmid (pU6-(BbsI)_Cbh-Cas9-T2A-mCherry; Addgene plasmid #64324) and 150 ng OOF plasmid (pBS SK mCherryROSAegfp; Addgene plasmid #54322) according to manufacturer’s protocol and incubated at 37°C for 48 hours before flow cytometry read-out. gRNAs were considered working when at least 33% of the transfected cells were GFP^+^.

### Flow Cytometry

Cells were stained with LIVE/DEAD^®^ Fixable Red Dead Cell Stain Kit (L34972, Thermo Fisher), LIVE/DEAD^®^ Fixable Near-IR Dead Cell Stain Kit (L34976, Thermo Fisher) or Propidium Iodide Staining Solution (PI, 556463, BD Biosciences) according to the manufacturer’s instructions for assessment of viability. Cell surface staining was performed in MACS buffer [PBS (17-516F, Lonza) + 0.5% BSA (A2153-100G, Sigma-Aldrich) + 2 mM EDTA (15575-038, Invitrogen)] by incubating fluorochrome-conjugated antibodies for 20 minutes at 4°C. Afterwards, cells were fixed and permeabilized using eBioscience™ FOXP3/Transcription Factor Staining Buffer set (00-5523-00, Invitrogen) according to manufacturer’s protocol. Intracellular staining was performed in Perm buffer by incubating antibodies for 30 minutes at 4°C. For intracellular cytokine staining, cells were stimulated with 50ng/ml phorbol12-myristate13-acetate (PMA) and 250ng/ml Ionomycin (Sigma) in the presence of GolgiPlug (BD) for 5 hours. Flow cytometry analyses were performed using LSRFortessa X-20 (BD Biosciences) and FlowJo™ (BD Biosciences). Antibodies used were CD4 – APC-Cy7 (557871, BD Biosciences), CD8 – APC (17-0088-73, eBioscience), CD25 – PE-Cy7 (557741, BD Biosciences), CD127 – PerCP-Cy5.5 (351322, Biolegend), B2M – FITC (316304, Biolegend), CD126 – PE (352804, Biolegend), FOXP3 – PE (320108, Biolegend), FOXP3 – AF700 (56-4776-41, eBioscience), Helios Alexa Fluor 488 (563950, BD Biosciences), TIGIT Brilliant Violet 605 (372712, Biolegend), CD39 FITC (328206, Biolegend), CTLA4 PE (555853, BD Biosciences), IL-2 APC (17-7029-82, eBioscience), IFNγ FITC (11-7319-82, eBioscience), IL-10 PE (559330, BD Biosciences), IL-17A PerCP-Cy5.5 (45-7179-42, eBioscience).

### Suppression Assay

Treg ability to suppress T cell proliferation was assessed as previously described (36, 37). In brief, allogeneic PBMCs (100.000 cells/well) were stained with CellTrace™ CFSE Cell Proliferation Kit (C34554, Thermo Fisher) at 1 µM and cultured with Tregs (in different Treg : PBMC ratios: 1:2, 1:4 and 1:8) in 96-well U-bottom plates in X-vivo + 5% FBS. Cells were stimulated using Treg Suppression Inspector beads (130-092-909, Miltenyi Biotec) on a 1:1 bead to cell ratio and cultured for 4 days before FACS analysis. After incubation, cells were stained for flow cytometry analysis as mentioned above and acquired on a BD LSRFortessa. Briefly, cells were stained with LIVE/DEAD^®^ Fixable Red Dead Cell Stain Kit to exclude dead cells. Cells were then extracellularly stained for CD4 and CD8 expression, fixed and permeabilized using eBioscience™ FOXP3/Transcription Factor Staining Buffer set and lastly stained intracellularly for FOXP3 expression. Treg suppression capacity was assessed based on PBMC proliferation extracted from positive CellTrace™ CFSE staining on both CD4 and CD8 positive populations, allowing a clear exclusion of Tregs from PBMC cells.

### IL-6 Pre-Incubation

Tregs were isolated from buffy coats as mentioned before and incubated in X-vivo + 5% FBS the presence of 1 µg/mL of plate-bound anti-CD3 (555329, BD Biosciences), 1 µg/mL of soluble anti-CD28 (555725, BD Biosciences) and 25 U/mL IL-2 (11147528001, Sigma-Aldrich) in 96-well U-bottom plates at 5x10^4^ cells per well for 24 hours. Where indicated, Tregs were further incubated in the presence of 25 ng/mL IL-6 (206-IL-010, R&D Systems).

### Phosflow

50.000 cells/well were plated in V-bottom 96-well plates and incubated for 2 hours at 37°C. After incubation, 100 U/ml IL-2 (11147528001, Sigma-Aldrich) or 50 ng/ml IL-6 (206-IL-010, R&D Systems) was added to each well and cells were incubated at 37°C for 15 minutes. Subsequently, cells were fixed using BD Cytofix™ Fixation Buffer (554655, BD Biosciences) according to manufacturer’s protocol (10 minutes at 37°C). Next, cells were washed in PBS + 0.5% BSA and permeabilized using BD Perm III buffer (558050, BD Biosciences) according to manufacturer’s instructions (30 minutes on ice). Then cells were washed twice in PBS + 0.5% BSA and stained in PBS + 0.5% BSA with pSTAT5 – Pacific Blue (560311, BD Biosciences) or pSTAT3 – FITC (651019, Biolegend) and analyzed using LSRFortessa X-20 (BD Biosciences) and FlowJo™ (BD Biosciences).

### Sequencing Analysis

Genomic DNA was extracted from Tregs with QIAamp DNA blood mini kit (51104, Qiagen) according to manufacturer’s protocol. Specific gene fragments were amplified using HotStarTaq Master Mix Kit (203443, Qiagen) according to manufacturer’s protocol and gene specific primers listed in **Table S2**. In a thermal cycler, the following PCR program was used: 15 minutes at 95°C, 40 cycles of 30 seconds at 95°C, 30 seconds at 65°C and 1 minute at 72°C, followed by 10 minutes at 72°C. PCR products were loaded on a 1% agarose gel and extracted using Nucleospin PCR and Gel Clean-up kit (740609.250, Macherey-Nagel). Sanger sequencing was performed at LGC Genomics GmbH (Berlin, Germany) and the data were analyzed using the ICE analysis tool (Synthego).

### Statistical Analysis

Statistical analyses were performed using GraphPad Prism 8.2 software (GraphPad Software). Error bars represent mean ± SD. Results were compared using two-tailed unpaired and paired t tests and one-way ANOVA if the data were normally distributed. Wilcoxon and Kruskal-Wallis tests were used as non-parametric tests. Normality was assessed using Shapiro-Wilk tests. For all experiments, significance was defined as *p ≤ 0.05, **p ≤ 0.01, ***p ≤ 0.001 and ****p ≤ 0. 0001.

## Results

### Treg Expansion Under Maintenance of FOXP3 Expression and Suppressive Capacity

**Figure 1:**
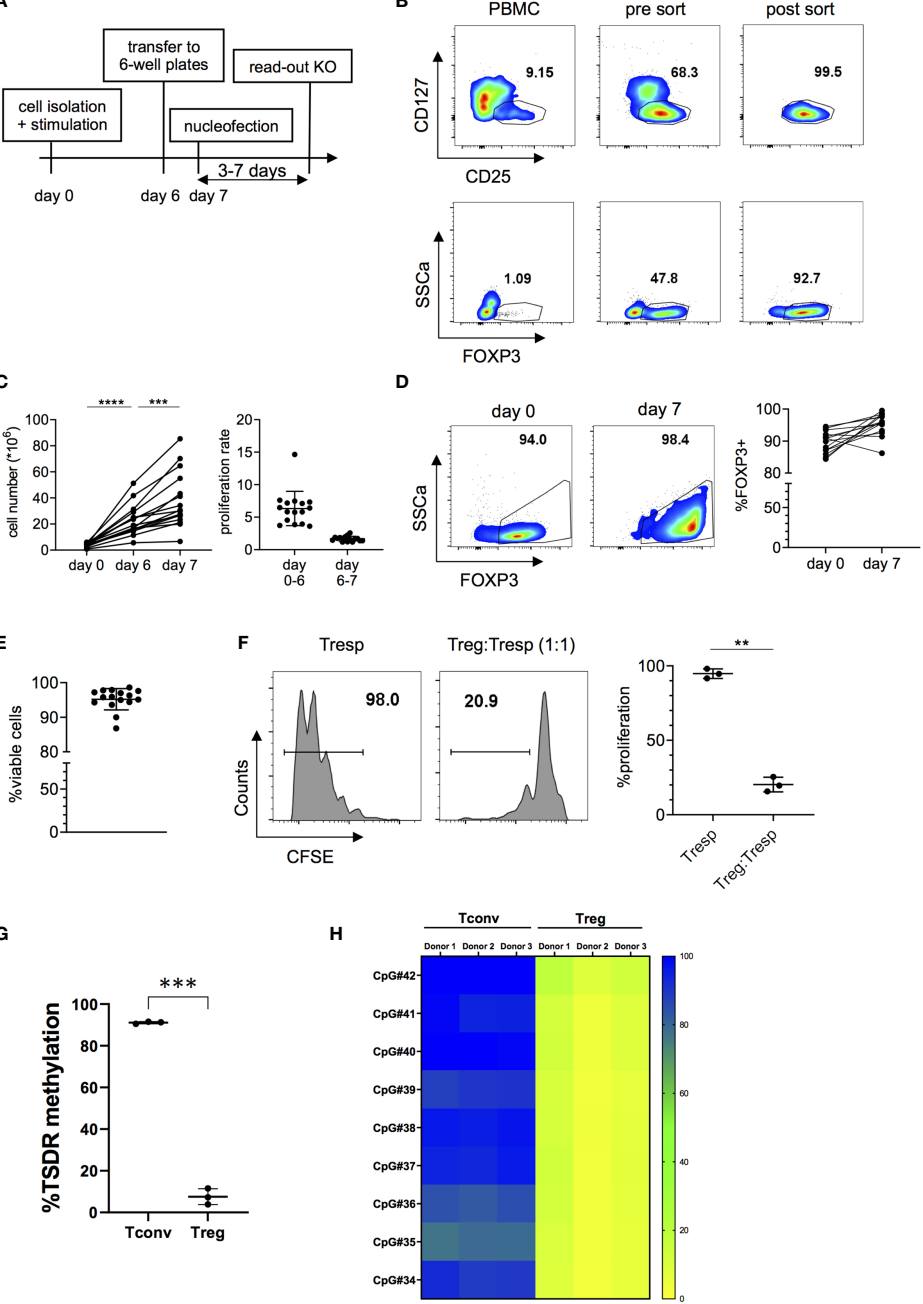
Isolation and *in vitro* expansion of human Tregs. **(A)** Schematic overview of the cell expansion procedure. Tregs are stimulated *in vitro* for six days with anti-CD3 and anti-CD28 antibodies in the presence of IL-2 and then transferred to 6-well plates 24 hours prior to nucleofection in the absence of TCR stimulation. Read-out of the KO was performed 3 to 7 days after nucleofection. **(B)** Representative FOXP3 expression before and after FACS sorting CD4^+^CD25^+^CD127^-^ cells. **(C)** Cell numbers (left panel) and proliferation rate (right panel) of *in vitro* expanded Tregs (n = 16 independent donors). Data is depicted as mean ± SD. **(D)** FOXP3 expression before stimulation and after seven days in culture of one representative donor (left panel) and 16 independent donors (right panel). **(E)** Cell viability after seven days in culture (n = 16 independent donors). Error bars represent mean ± SD. **(F)** Suppressive capacity of Tregs after seven days of *in vitro* culture, measured as the ability of Tregs to suppress CD4^+^ T cell (Tresp) proliferation. FACS data of one representative donor (left panel) and cumulative data of 3 independent donors (right panel) are shown. **(G, H)** DNA methylation pattern of the Treg-Specific Demethylation Region (TSDR) assessed in 7 day-*in vitro* expanded CD4^+^CD25^-^CD127^+^ Tconv and CD4^+^CD25^+^CD127^-^ Treg cell subsets. **(G)** Average methylation rate for 9 CpG sites of the TSDR. Each dot denotes a donor (n =3 different donors matched for Tconvs and Tregs). **(H)** In the heatmap plot, each box represents the percentage of methylation of a single CpG residue for each sample (n= 3 matched donors for Tconvs and Tregs). Bar colors designate: yellow: 0% methylation; green: 50% methylation; blue: 100% methylation. Error bars represent mean ± SD. Significance was calculated using one-way ANOVA **(C)** or two-tailed paired t test **(F, G)**. **p ≤ 0.01, ***p ≤ 0. 001 and ****p ≤ 0.0001.

In order to generate sufficient cell numbers for functional experiments with highly pure Tregs, we optimized an *in vitro* expansion protocol for human Tregs, maintaining high viability and functionality. Tregs were isolated from peripheral blood mononuclear cells (PBMC) and stimulated for six days with plate-bound anti-CD3 and soluble anti-CD28 mAbs in the presence of IL-2. Since it has been observed that high cell densities negatively affect transfection efficiency (38), cells were rested for 24 hours prior to transfection in 6-well plates in the presence of IL-2 but without further TCR stimulation. A schematic representation of this protocol is shown in **Figure 1A**. Tregs were isolated from PBMCs using CD25 microbeads and subsequently FACS-sorted as CD4^+^CD25^+^CD127^-^ cells (**Figure 1B** and **Figure S1**). Expression of Treg-associated markers FOXP3, Helios, TIGIT, CD39 and CTLA4, assessed after sorting, confirms the high purity of isolated Tregs (**Figure 1B** and **Figure S2**). Tregs were then expanded *in vitro* for 6 days and were rested for one more day before transfection (**Figure 1C**). Importantly, expanded Tregs maintained high cell viability and expression of Treg-associated markers after expansion (**Figures 1D, E** and **Figure S2**). Moreover, *in vitro* expanded Tregs preserved their suppressive capacity (**Figure 1F**) and a demethylated TSDR profile (**Figures 1G, H**). Overall, these data indicate that our protocol efficiently induces proliferation of highly pure *ex vivo* isolated Tregs with sufficient proliferation rates in a short timeframe and without loss of functionality, FOXP3 expression or Treg stability. Importantly, the protocol did not lead to expansion or outgrowth of contaminating effector cells as demonstrated by the phenotype and low TSDR methylation in the expanded Treg product.

### Highly Efficient RNP-Based Genome Editing in Human Tregs

**Figure 2:**
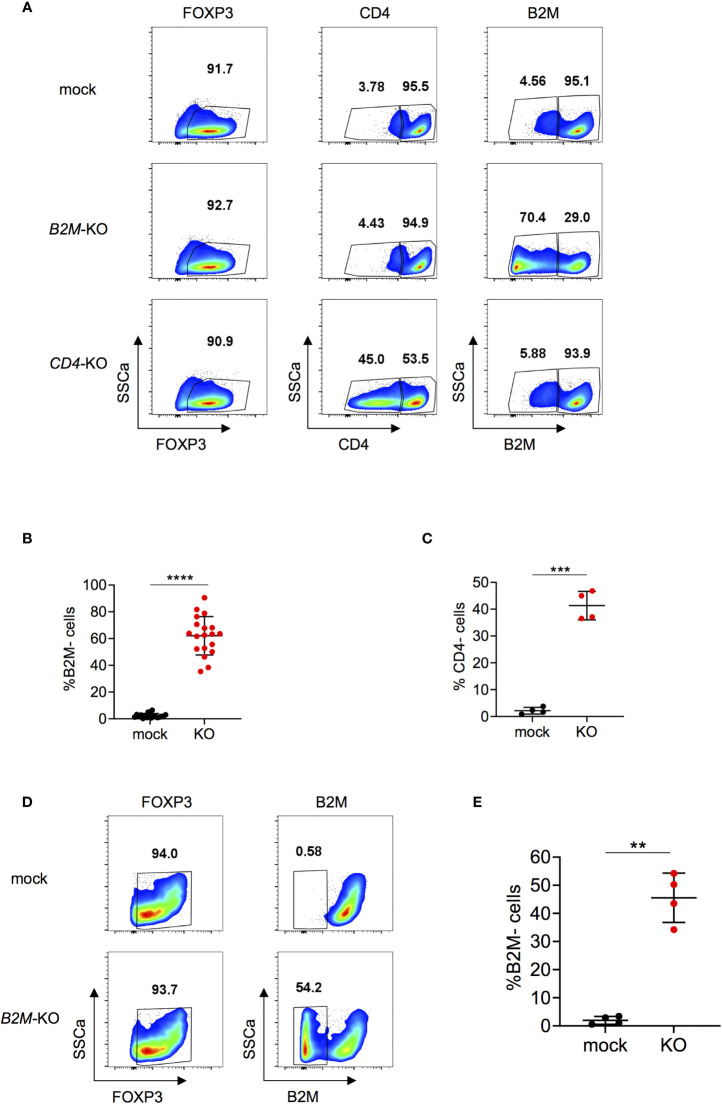
*B2M* and *CD4-*KO in human Tregs. **(A)** Human Tregs were cultured for 7 days *in vitro* prior gene editing (following protocol described in **Figure 1A**). FOXP3, CD4 and B2M expression were analyzed in mock, *B2M*-KO and *CD4*-KO Tregs that were harvested 5 days after RNP nucleofection. FOXP3 plots are pre-gated on living cells. Plots showing CD4 and B2M expression are pre-gated on living FOXP3^+^ cells. Data are from one representative experiment. **(B)** B2M protein expression was studied in *B2M-KO* Tregs 3 to 7 days after nucleofection (n = 19 independent donors). Error bars represent mean ± SD. **(C)** CD4 protein expression was studied in *CD4-KO* Tregs 3 to 5 days after nucleofection (n = 4 independent donors). Error bars represent mean ± SD. **(D, E)** Tregs were stimulated for 24 hours prior gene editing. FOXP3 and B2M expression were analyzed in mock or *B2M*-KO Tregs 4 days after RNP nucleofection. Plots showing FOXP3 expression are pre-gated on living cells. B2M plots are pre-gated on living FOXP3^+^ cells. Data are from one representative experiment **(D)** and 4 independent donors **(E)**. Error bars represent mean ± SD. Significance was calculated using two-tailed paired Wilcoxon test **(B)** or by a two-tailed paired t test (C,E). **p ≤ 0.01, ***p ≤ 0. 001, ****p ≤ 0.0001.

Next, expanded Tregs were used for RNP-based genome editing. To assess feasibility and efficacy of the KO procedure in Tregs, we chose to KO beta-2-microglobulin (*B2M*) and *CD4* genes. B2M is a component of the major histocompatibility (MHC) class I molecules and is present on all nucleated cells except for red blood cells (39). CD4 is a membrane-bound glycoprotein that is expressed on helper T cells (40). *B2M*-KO reached efficiencies up to over 90% depending on the donor and on average about 60% at protein level at the time points analyzed (**Figures 2A, B**). This is well in line with the representative 67% efficiency observed by Inference of CRISPR Edits (ICE) analysis (**Figure S3**). Briefly, ICE uses Sanger sequencing data for analysis of CRISPR KO efficiencies on DNA level (41). Efficacy for *CD4-*KO reached about 40% for CD4 expression at protein level (**Figures 2A, C**). Treg KO efficiencies were similar for B2M and slightly lower for CD4 compared to genome editing in total CD4^+^ T cells (**Figures 2B, C** and **Figure S4**). *B2M*-KO did not affect viability compared to mock condition (**Figure S5A**). Moreover, FOXP3 expression was stable in both mock and KO cells, indicating that the gene editing protocol does not compromise cell integrity (**Figure S5B**). Furthermore, re-stimulation after nucleofection did not affect viability, FOXP3 expression nor KO efficiency (**Figures S6A–D**). Altogether, these data indicate that *B2M* and *CD4* can be efficiently knocked-out in Tregs without compromising FOXP3 expression or viability, and KO phenotype is conserved upon re-stimulation with diverse TCR stimulating conditions.

Since *in vitro* cell expansion does not recapitulate the physiology of *ex vivo* Tregs, certain studies may require gene editing of non-expanded Tregs. Therefore, we investigated whether Tregs could be gene edited under short-term stimulation prior to RNP nucleofection. Using these conditions we achieved *B2M*-KO efficiencies of, on average, 45% (**Figures 2D, E**), with no effect on viability (**Figure S7A**) or FOXP3 expression (**Figure S7B**) compared to mock controls. Thus, our data demonstrates that human Tregs can be efficiently genome edited, also by using a short-term protocol.

### Efficient Multiplexing in Human Tregs Without Compromising Cell Integrity

**Figure 3:**
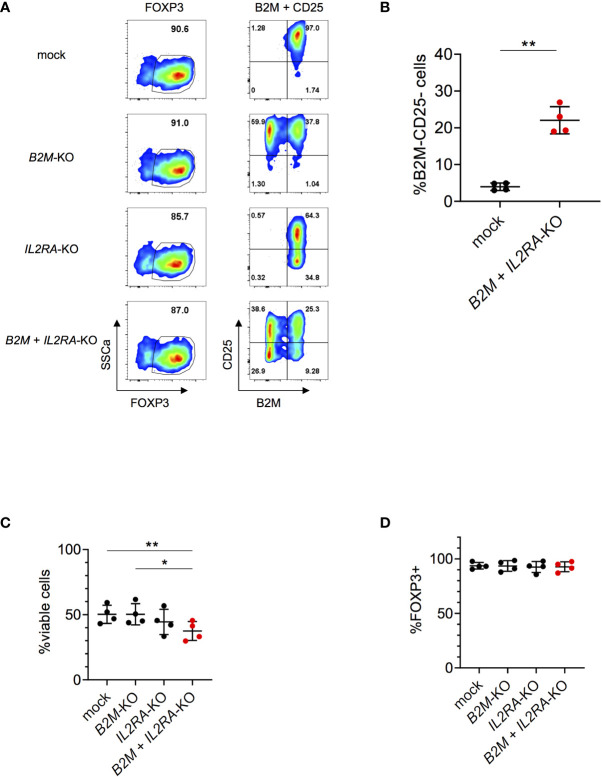
Efficient multiplexing of *B2M* and *IL2RA-*KO in human Tregs. **(A)** Human Tregs were cultured *in vitro* for 7 days prior gene editing (following protocol described in **Figure 1A**). FOXP3, CD25 and B2M expression were analyzed in mock, *B2M*-KO, *IL2RA*-KO and double KO Tregs that were harvested 4 days after RNP nucleofection. FOXP3 plots are pre-gated on living cells. Plots showing B2M/CD25 expression are pre-gated on living FOXP3^+^ cells. Data are from one representative experiment out of 4. **(B)** Percentages of double KO of *B2M* and *IL2RA* in human Tregs were analyzed 4 days after nucleofection (n = 4 independent donors). Error bars represent mean ± SD. **(C)** Viability of *B2M* and *IL2RA* single and double KO Tregs was measured 4 days after nucleofection (n = 4 independent donors). Error bars represent mean ± SD. **(D)** FOXP3 expression of *B2M* and *IL2RA* single and double KO Tregs (pre-gated on living cells) was measured 4 days after nucleofection (n = 4 independent donors). Error bars represent mean ± SD. Statistical significance was calculated by a two-tailed paired t test **(B)** and one-way ANOVA **(C, D)**. *p ≤ 0.05, **p ≤ 0.01.

We next tested the applicability and efficiency of our gene editing protocol for multiplexing, knocking-out multiple genes at once. When targeting *B2M* and *IL2RA* (encoding for the α-chain of the IL2 receptor; CD25) in parallel, we were able to generate about 20-30% double KO by co-transfection of both sgRNAs simultaneously (**Figures 3A, B**). Multiplexing slightly affected viability (**Figure 3C**) and did not affect FOXP3 expression (**Figure 3D**), assessed four days after nucleofection. KO efficiencies were 49.8% and 25.4% of the single KOs of *B2M* and *IL2RA* respectively, when measured at protein level (**Figure S8**). Overall, these data show that multiplexing in human Tregs can be performed in an efficient way with limited effects on cell viability and cell integrity, offering possibilities to target combinations of genes to study Treg function and potentially improve Treg-based immunotherapy.

### CD25-KO in Tregs Compromises Suppressive Capacity and STAT5 Phosphorylation

**Figure 4:**
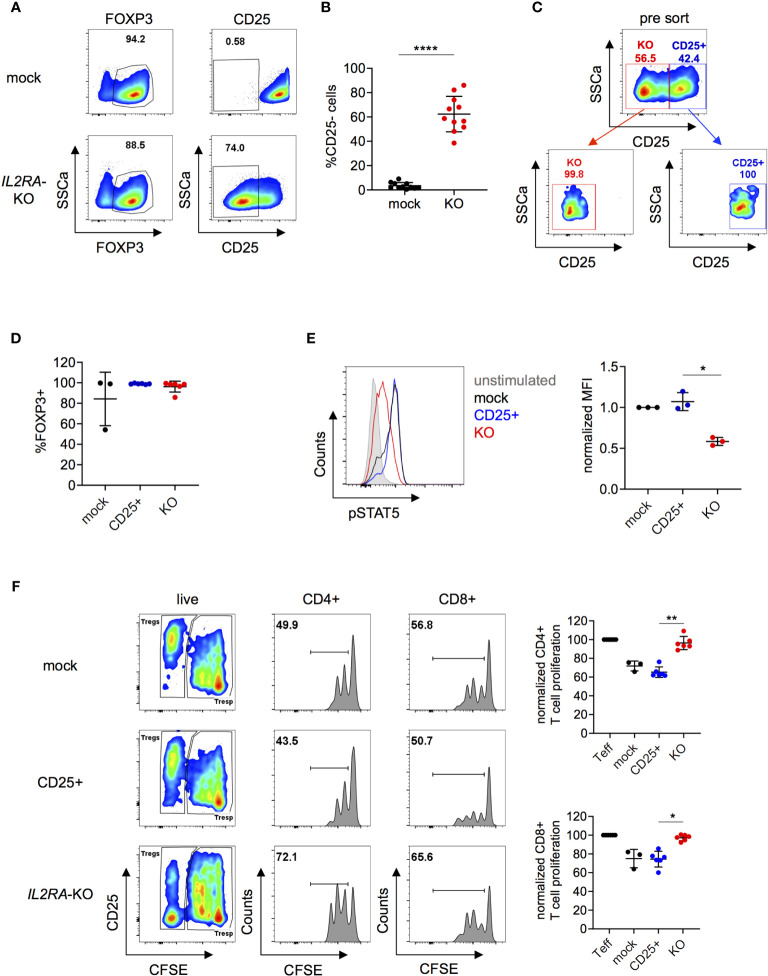
Knock-out of *IL2RA* in human Tregs impairs suppressive capacity and IL-2-mediated signaling. Human Tregs were cultured *in vitro* for 7 days prior gene editing (following protocol described in **Figure 1A**). **(A)** FOXP3 and CD25 expression were analyzed in mock and *IL2RA*-KO Tregs that were harvested 4 days after RNP nucleofection. FOXP3 plots are pre-gated on living cells and CD25 plots are pre-gated on living FOXP3^+^ cells. Data are from one representative experiment. **(B)** CD25 expression was studied in mock and *IL2RA-KO* Tregs 4 to 7 days after nucleofection. n = 11 independent donors. Error bars represent mean ± SD. **(C)** RNP-transfected cells were FACS-sorted for CD25 expression and as living (PI^-^) CD4^+^CD127^-^ cells. Top panel displays pre-sorted cells and bottom panels represent re-analysis post sorting. **(D)** FOXP3 expression after CD25-sorting in mock (n = 3), CD25^+^ (n = 6) and *IL2RA*-KO (n = 6) Tregs. Error bars represent mean ± SD. **(E)** STAT5 phosphorylation was studied in CRISPR-edited Tregs. FACS plot of one representative experiment (left panel) and combined data of three independent donors (right panel). Error bars represent mean ± SD. MFI is normalized over IL-2-stimulated mock. **(F)** Suppressive capacity of *IL2RA*-KO Tregs was measured by their ability to suppress T cell proliferation *in vitro* (ratio 1:2). Left panels show FACS plots of one representative donor. Cells are pre-gated for viability (all columns) and CD4 or CD8 expression (middle and right column, respectively). Right panels show CD4^+^ and CD8^+^ T cell proliferation, displayed by dilution of the cell proliferation dye CFSE. Proliferation is normalized over T cell proliferation in the absence of Tregs (n = 3-6 independent donors). Error bars represent mean ± SD. Significance was calculated by two-tailed paired t test **(B)**, Kruskal-Wallis test **(D, F)** or one-way ANOVA **(E)**. *p ≤ 0.05, **p ≤ 0.01. ****p ≤ 0.0001.

Next, we investigated if the suppressive capacity of Tregs could be altered by knocking out *IL2RA*, as previously demonstrated in murine Tregs (7, 42–45). CD25 forms, together with CD122 and CD132, a fully functional IL-2 receptor that can activate the transcription factor STAT5 (45). Roth et al. described a family with monogenic immune disease caused by a heterozygous mutation in *IL2RA* leading to ablation of CD25 expression and decreased STAT5 phosphorylation in Tregs. Furthermore, Tregs from those patients showed decreased suppressive capacity (46). *IL2RA-*KO in Tregs following our protocol, reached efficiencies up to over 80% depending on the donor and on average 60% on protein level (**Figures 4A, B**). Mock, CD25^+^ and *IL2RA*-KO cells were FACS sorted with high purity four days after nucleofection as living cells and based on CD25 expression (**Figure 4C**). FOXP3 expression did not differ between mock, CD25^+^ and *IL2RA*-KO (**Figure 4D**). *IL2RA*-KO Tregs also maintained similar Helios and TIGIT expression as mock Tregs, whereas a downregulation in CTLA4 expression was observed in *IL2RA*-KO Tregs (**Figure S9A**). These data are consistent with previous findings that reported no changes in Helios expression in patients with an *IL2RA* null mutation (47), and CTLA4 downregulation in *IL2RA*-KO murine Tregs (48). Moreover, we could not detect major changes in expression of cytokines such as IL-2, IL-10, IFNγ or IL-17A in *IL2RA*-KO Tregs (**Figure S9B**). DNA sequencing analysis of sorted KO cells revealed a KO score of 70%, indicating that both homozygous as well as heterozygous mutations cause loss of CD25 protein expression (**Figure S10A**). Importantly, *IL2RA*-KO Tregs had significantly decreased STAT5 phosphorylation upon IL-2 stimulation, proving functional KO of *IL2RA* targeted cells (**Figure 4E**). Moreover, *IL2RA-*KO Tregs had significantly blunted suppressive capacity compared to control Tregs, measured as their ability to inhibit proliferation of CD4^+^ and CD8^+^ T effector cells in suppression assays (**Figure 4F**). These data highlights the importance of CD25 as a functional component in *in vitro* suppression assays, comparable to published data on murine Tregs (7). Overall, the obtained results demonstrate that our gene editing protocol does not compromise cell integrity and that gene-edited cells can be efficiently used for functional downstream applications.

### Deletion of CD126 in Human Tregs Impairs IL-6-Mediated Signaling

**Figure 5:**
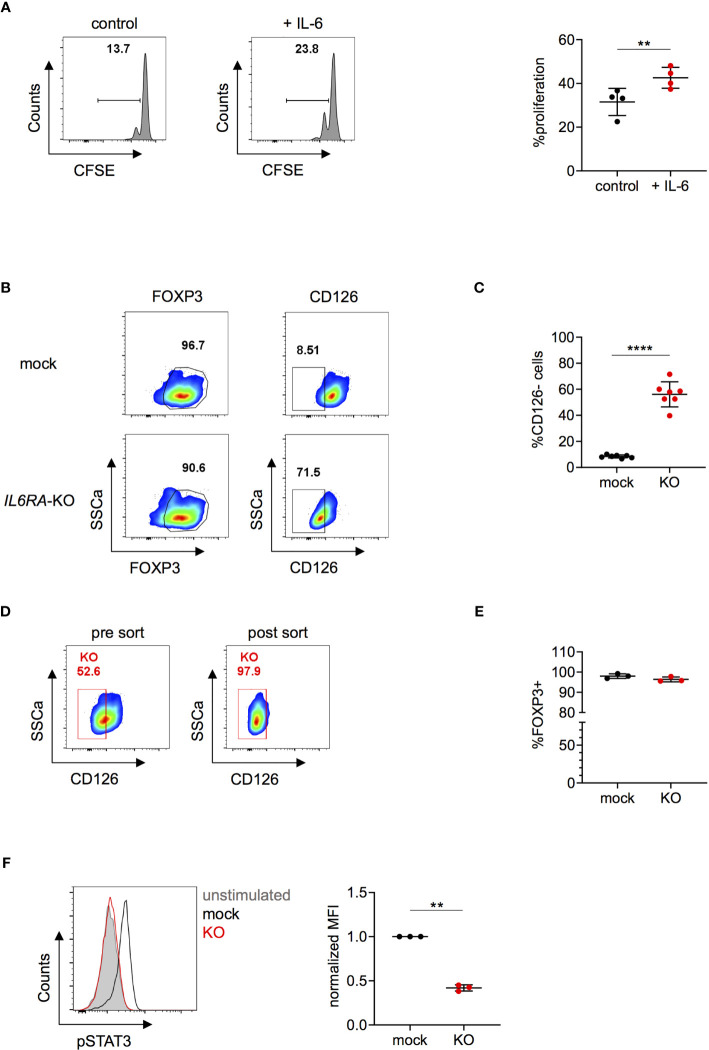
Knock-out of *IL6RA* in human Tregs impairs IL-6-mediated signaling. **(A)** Tregs were incubated without (control) or with IL-6 for 24 hours and subsequently cultured in suppression assays with CFSE-labeled PBMCs. FACS plots of one representative experiment (left panel) and data combining 4 independent donors (right panel). Error bars represent mean ± SD. **(B–F)** Human Tregs were cultured *in vitro* for 7 days prior gene editing (following protocol described in **Figure 1A**). **(B)** FOXP3 and CD126 expression were analyzed in mock and *IL6RA*-KO Tregs that were harvested 5 days after RNP nucleofection. FOXP3 plots are pre-gated on living cells and CD126 plots are pre-gated on living FOXP3^+^ cells. Data are from one representative experiment. **(C)** CD126 expression was studied in mock and *IL6RA-KO* Tregs 4 to 7 days after nucleofection in n = 7 independent donors. Error bars represent mean ± SD. **(D)** RNP-transfected cells were FACS-sorted for CD126 expression and as living (L/D nearIR^-^) CD4^+^CD25^+^CD127^-^ cells. Left panel represents pre-sorted cells and right panel shows re-analysis post sorting. **(E)** FOXP3 expression in CD126^-^ sorted cells (n = 3). Error bars represent mean ± SD. **(F)** STAT3 phosphorylation was analyzed in CRISPR-edited Tregs. FACS plot of one representative experiment (left panel) and data from 3 independent donors (right panel). Error bars represent mean ± SD. MFI is normalized over IL-6-stimulated mock. Statistical significance was calculated by two-tailed t test. **p ≤ 0.01, ****p ≤ 0.0001.

Several clinical trials indicate that tocilizumab might be an effective therapy to ameliorate disease severity in COVID-19 patients (24–28). Since CD126 is expressed on both CD4^+^CD25^-^ conventional T cells as well as on Tregs (49), tocilizumab could act on both cell types and it was recently shown that tocilizumab treatment also affects the transcriptional signature of Tregs in COVID-19 patients (50). IL-6 is a well known regulator of the Th17 and Treg balance (21, 51) and murine CD126^+^ Tregs have defective suppressive function whereas CD126^-^ Tregs exerted superior stability in an inflammatory context *in vivo* (20). However, the effects of IL-6 on human CD4^+^ T cells are less established. Ferreira et al. described a subset of human Tregs that are highly suppressive *in vitro* and can be characterized by high IL-6 receptor expression that potentially could relate to Treg instability in the presence of IL-6-associated inflammation *in vivo* (52). Further, it has been demonstrated that supplementation of IL-6 to *in vitro* suppression assays impairs the suppressive function of human Tregs (19). However, these observations might be the result of effects of IL-6 on responder cells rather than on Tregs. In order to test the direct effects of IL-6 on human Tregs, we pre-incubated Tregs for 24 hours with IL-6 before setting up an *in vitro* suppression assay and observed decreased suppressive ability compared to controls (**Figure 5A**). To further investigate the function of CD126, we knocked out *IL6RA* in Tregs using our protocol. We reached KO efficiencies over 70% depending on the donor and on average of about 55% on protein level, as measured by FACS for CD126 expression (**Figures 5B, C**). For downstream analysis, *IL6RA*-KO cells were purified by FACS-sorting (**Figure 5D**). ICE analysis of sorted KO cells confirmed a high KO score of 90%, indicating that most of the KO sorted cells are homozygous for a mutation (**Figure S10B**). FOXP3 expression was not affected by *IL6RA-*KO (**Figure 5E**) and *IL6RA*-KO Tregs also maintained similar Helios and TIGIT expression (**Figure S11A**), in line with previous data showing similar Helios expression between TIGIT^+^IL-6R^high^ and TIGIT^+^IL-6R^low^ human memory Tregs (52) or between gp130^high^ or gp130^low^ human memory Tregs (gp130 being part or the IL-6R complex) (19). Moreover, *IL6RA*-KO Tregs did not show major differences in IL-2, IL-10, IFNγ or IL-17A expression compared to mock Tregs at different time points after RNP nucleofection (**Figures S11B, C**). IL-6 induced STAT3 signaling could activate a Th17-like phenotype in Tregs while destabilizing their function (15, 17, 21). In order to test functional consequences of *IL6RA*-KO we thus examined STAT3 phosphorylation in targeted Tregs. Importantly, upon IL-6 stimulation, *IL6RA*-KO cells had significantly lower phosphorylation of STAT3 compared to mock-transfected Tregs (**Figure 5F**). Overall, these data further demonstrate that human Treg function could be directly affected by IL-6 and that targeting of CD126 may prevent IL-6-mediated instability.

## Discussion

Here, we describe a fast and efficient method for generating functional KOs in human Tregs, based on CRISPR/Cas9 technology wherein editing components are delivered as RNP. CRISPR/Cas has dramatically changed the field of genetic engineering and made possible the use of genome edited-cells as cellular therapies. CRISPR-mediated gene KO in primary human T cells is well described and reaches high efficiencies (34, 46, 53–55). More recently, studies have described gene targeting in different CD4^+^ T cell subsets, such as Th1, Th2 and Tregs (55–57). However, methods for specific genome editing in human Tregs are not well established due to their scarcity in peripheral blood and difficulty to culture *in vitro*, highlighting the need for more rapid and effective protocols.

While most protocols for Treg culture include the use of synthetic beads coated with anti-CD3 and anti-CD28 mAbs (55, 56, 58, 59), we show that Tregs can be efficiently targeted after being *in vitro* expanded in a bead-free system by the use of anti-CD3/CD28 mAbs in the presence of IL-2. Importantly, the expanded cells maintained the typical Treg characteristics, such as FOXP3, Helios and CTLA4 expression, TSDR demethylation and high suppressive capacity. The use of RNPs eliminates the need for viral transduction and it is a safer option for potential clinical application. It is known that optimal KO conditions may vary between cell types and activation status. We demonstrate the possibility of KO multiple gene targets in human Tregs, such as *B2M* and *CD4*, with efficiencies comparable to total CD4^+^ T cells and with minimal effect on cell viability or FOXP3 expression. Moreover, KO was also possible at different stages of Treg activation (*in vitro* stimulation for 24 hours or 7 days). Importantly, KO phenotype is maintained after TCR re-stimulation, allowing prolonged Treg expansion to obtain a sufficient number of cells for following applications. In addition, targeting multiple genes simultaneously would benefit research of complex gene interactions and may well be necessary to create the most efficient Treg cellular product. Here, we show that multiplexing is possible following our described genome editing protocol. We created *B2M* and *IL2RA* double KO Tregs, that maintained high viability and FOXP3 expression.

Importantly, we show that genome edited Tregs could be used in downstream applications – such as suppression assays and modulation of cytokine induced signaling – in order to further elaborate the function of the targeted genes. Tregs express high levels of CD25, that could effectively deprive effector T cells of IL-2 (7, 42–45). We generated *IL2RA-KO* Tregs and corroborated that human Tregs lacking CD25 were less suppressive compared to controls. Another explanation for the loss of suppressive function may be that *IL2RA*-KO Tregs do not survive because of impaired IL-2 signaling, as demonstrated by reduced STAT5 signaling.

We further knocked out the α-subunit of the IL-6 receptor (CD126) in human Tregs. IL-6 is a well-known destabilizer of Tregs (15, 17–21), present at high levels in numerous immune-related diseases including SLE, MS, RA [reviewed in (22)] and also in critically ill COVID-19 patients (23). IL-6 activates the transcription factor STAT3, which could downregulate FOXP3 while promoting the expression of the Th17 cell-associated transcription factor RORγt (17). Our data demonstrated that human Tregs pre-activated in the presence of IL-6 had impaired suppressive activity *in vitro*, indicating direct effects of IL-6 on Treg function. In line with this, we demonstrated that *IL6RA*-KO Tregs do not activate STAT3 in response to IL-6 stimulation. These data suggest that inhibiting *IL6RA* expression could improve stability of Treg cell products intended to be used as a cellular therapy for autoimmune and infectious diseases like COVID-19. Although the role of Tregs in COVID-19 is not understood yet, accumulating data indicate that changes in Tregs are associated with severe disease (29, 60–67). The potential of Tregs in the context of COVID-19 is being discussed (68, 69) and the therapeutic value of Treg products is currently under investigation (NCT04482699, NCT04468971). Of note, a recent case report indicated already its applicability and potential positive effects (70). Moreover, tocilizumab, a mAb against CD126, is currently being used for the treatment of RA and is being explored for the treatment of severely ill COVID-19 patients (24–28). Given CD126 expression pattern, tocilizumab likely acts not only on T effector cells but also on Tregs, possibly by preventing Treg instability. More research in order to unravel the underlying mechanisms of action of tocilizumab and of IL-6 on the T cell balance is thus required.

In summary, our study reports a robust and efficient technique to rapidly generate gene KO in human Tregs, without compromising viability or FOXP3 expression. Tregs are being explored for the treatment of inflammatory disorders such as autoimmunity, transplantation and infection diseases. Data from clinical trials have shown feasibility and safety of Treg cell therapy (71–73), although its efficacy is not conclusive yet. T cell genome engineering has revolutionized the field of adoptive-T cell therapy and could also well improve the efficacy and specificity of cellular therapies using Tregs. In this regard, cytokines also play a key role in regulating Treg function. Our data demonstrate that human Treg function can be modulated by knocking out diverse cytokine receptor genes, such as *IL2RA* and *IL6RA*. Since Treg function is likely being affected by high IL-6 levels under inflammatory conditions such as in autoimmunity or infectious diseases like COVID-19 *in vivo*, CD126 might be a potential target to enhance Treg stability and function in pro-inflammatory environments. However, future studies in *in vitro* and *in vivo* model systems have to define the specific functional characteristics and long-term stability of genome edited Tregs.

## Data Availability Statement

The original contributions presented in the study are included in the article/**Supplementary Material**. Further inquiries can be directed to the corresponding author.

## Ethics Statement

The studies involving human participants were reviewed and approved by institutional review board UHasselt (CME2019/042 and CME2016/629). The patients/participants provided their written informed consent to participate in this study.

## Author Contributions

LVZ and RAH designed and performed experiments, analyzed and interpreted the data, and wrote the manuscript. BC-R and IH performed experiments and analyzed data. TM gave conceptual input. MK led and conceived the project, supervised experiments, interpreted data, and wrote the manuscript. All authors contributed to the article and approved the submitted version.

## Funding

MK was supported by the European Research Council (ERC) under the European Union’s Horizon 2020 research and innovation program (640116) and by a SALK-grant from the government of Flanders and by an Odysseus-grant of the Research Foundation Flanders, Belgium (FWO).

## Conflict of Interest

The authors declare that the research was conducted in the absence of any commercial or financial relationships that could be construed as a potential conflict of interest.

## Publisher’s Note

All claims expressed in this article are solely those of the authors and do not necessarily represent those of their affiliated organizations, or those of the publisher, the editors and the reviewers. Any product that may be evaluated in this article, or claim that may be made by its manufacturer, is not guaranteed or endorsed by the publisher.

## Supplementary Material

Supplementary Figure 1 Detailed gating strategy for FACS-sorting Tconvs and Tregs from freshly isolated PBMCs. **(A)** CD25-enriched PBMCs were sorted as living CD4^+^CD25^+^CD127^-^ Tregs (top panels CD25-enriched PBMC). Bottom panels show purity after sort (post sort Tregs). **(B)** CD25-depleted PBMCs were sorted as living CD4^+^CD25^-^CD127^+^ Tconvs (top panels CD25-depleted PBMC). Bottom panels show purity after sort (post sort Tconv).

Supplementary Figure 2 Detailed phenotypic characterization of freshly isolated and *in vitro* expanded Tconv and Tregs. Tconv and Tregs were stained for several Treg markers after isolation at **(A)** day 0 or **(B)** after 7 days of *in vitro* expansion. Tconv and Tregs were expanded using 10µg/ml anti-CD3 Ab, 1µg/ml anti-CD28 Ab and 1500IU/ml Proleukin. Helios, TIGIT, CD39 and CTLA4 are plotted against FOXP3 for one representative donor. **(C)** Data from 4-6 independent donors.

Supplementary Figure 3 Indel signature of *B2M*-KO in Tregs. Schematic representation of the *B2M* gene. Blue boxes indicate exons. The gRNA sequence (20 bp) is indicated by the left open black box and the PAM sequence (3 bp) is indicated by the right open black box. The dotted line and scissors indicate the Cas9 cutting site. The different KO sequences are listed, percentages indicate the relative contribution of each sequence. KO score is generated by ICE analysis and indicates the percentage of KO on genomic level.

Supplementary Figure 4 *CD4* and *B2M-KO* in human CD4^+^ T cells. CD4^+^ T cells were stimulated *in vitro* for 2 days prior RNP nucleofection. CD4 and B2M expression were analyzed in mock, *B2M*-KO and *CD4*-KO CD4^+^ T cells 3 days after nucleofection. Plots are pre-gated on living cells.

Supplementary Figure 5 Viability and FOXP3 purity of *B2M-KO* Tregs. Cell viability **(A)** and FOXP3 expression **(B)** were studied in mock and *B2M-KO* Tregs 3 to 7 days after nucleofection. n = 19 independent donors. Error bars represent mean ± SD. Statistical significance was calculated using a two-tailed paired Wilcoxon test.

Supplementary Figure 6 Re-stimulation of KO Tregs. Mock transfected and *B2M*-KO Tregs were re-stimulated using anti-CD3 and anti-CD28 antibodies in the presence of IL-2. **(A)** FOXP3 and B2M protein expression were studied 6 days after re-stimulation. Plots show cells pre-gated as living cells. Data are from one representative experiment. **(B–D)** B2M expression **(B)**, cell viability **(C)** and FOXP3 expression **(D)** were studied in re-stimulated Tregs 4 and 7 days after nucleofection in 3 independent donors. Error bars represent mean ± SD. Statistical significance was studied using two-tailed paired t test. *p ≤ 0.05.

Supplementary Figure 7 KO in short-term stimulated Tregs. Tregs were cultured *in vitro* with anti-CD3 and anti-CD28 antibodies and in the presence of IL-2 for 24h prior RNP nucleofection. Viability **(A)** and FOXP3 expression **(B)** were studied 4 days after nucleofection in n = 4 independent donors. Error bars represent mean ± SD. Statistical significance was studied using two-tailed paired t test.

Supplementary Figure 8 KO efficiency of *B2M* and *IL2RA* single KO controls from the multiplex experiment (**Figure 3**). Human Tregs were cultured for 7 days *in vitro* prior gene editing (following protocol described in **Figure 1A**). B2M **(A)** or CD25 **(B)** expression were analyzed in mock and single *B2M*-KO or *IL2RA*-KO Tregs. Data show protein expression 4 days after RNP nucleofection in n = 4 independent donors. Error bars represent mean with SD. Statistical significance was calculated by a two-tailed paired t test.*p ≤ 0.05, **p ≤ 0.01.

Supplementary Figure 9 Phenotype of *IL2RA*-KO Tregs. *IL2RA* was knocked out in human Tregs as described in **Figure 4**. 4 days after RNP nucleofection, Helios, TIGIT and CTLA4 expression were analyzed in MOCK and *IL2RA*-KO Tregs in 3 independent donors. Statistical differences were analyzed by two-tailed paired t test, *p ≤ 0.05 **(A)**. 4 days after RNP nucleofection, cells were examined for IL-10, IL-2, IL-17A and IFNγ expression by intracellular flow cytometry. Representative FACS plots (top panel) or data from 2 independent donors (bottom panel) are depicted **(B)**.

Supplementary Figure 10 Indel signatures of *IL2RA* and *IL6RA-*KO Tregs. Schematic representation of the *IL2RA* gene **(A)** and the *IL6RA* gene **(B)**. Blue boxes indicate exons. The gRNA sequence (20 bp) is indicated by the left open black box and the PAM sequence (3 bp) is indicated by the right open black box. The dotted line and scissors indicate the Cas9 cutting site. The different KO sequences are listed, percentages indicate the relative contribution of each sequence. KO score is generated by ICE analysis and indicates the percentage of KO on genetic level.

Supplementary Figure 11 Phenotype of *IL6RA*-KO Tregs. *IL6RA* was deleted in human Tregs as described in **Figure 5**. 5 days after RNP nucleofection, Helios and TIGIT expression were analyzed in MOCK and *IL6RA*-KO Tregs in 4 independent donors. Statistical differences were analyzed by two-tailed paired t test **(A)**. 5 days after RNP nucleofection, cells of 1-2 independent donors were re-stimulated *in vitro* with anti-CD3/CD28 mAbs and IL-2 for 3 days **(B)** or 7 days **(C)** and subsequently examined for IL-10, IL-2, IL-17A and IFNγ expression by intracellular flow cytometry.
