# Supplementary material for: Fast and Efficient Genome Editing of Human FOXP3+ Regulatory T Cells
Source: Front Immunol. 2021 Aug 2;12:655122. doi: 10.3389/fimmu.2021.655122 (PMC8365355; doi:10.3389/fimmu.2021.655122)
Supplement: Supplementary Figure 1 — Detailed gating strategy for FACS-sorting Tconvs and Tregs from freshly isolated PBMCs. (A) CD25-enriched PBMCs were sorted as living CD4+CD25+CD127- Tregs (top panels CD25-enriched PBMC). Bottom panels show purity after sort (post sort Tregs). (B) CD25-depleted PBMCs were sorted as living CD4+CD25-CD127+ Tconvs (top panels CD25-depleted PBMC). Bottom panels show purity after sort (post sort Tconv). [file DataSheet_1.pdf]

Figure S1

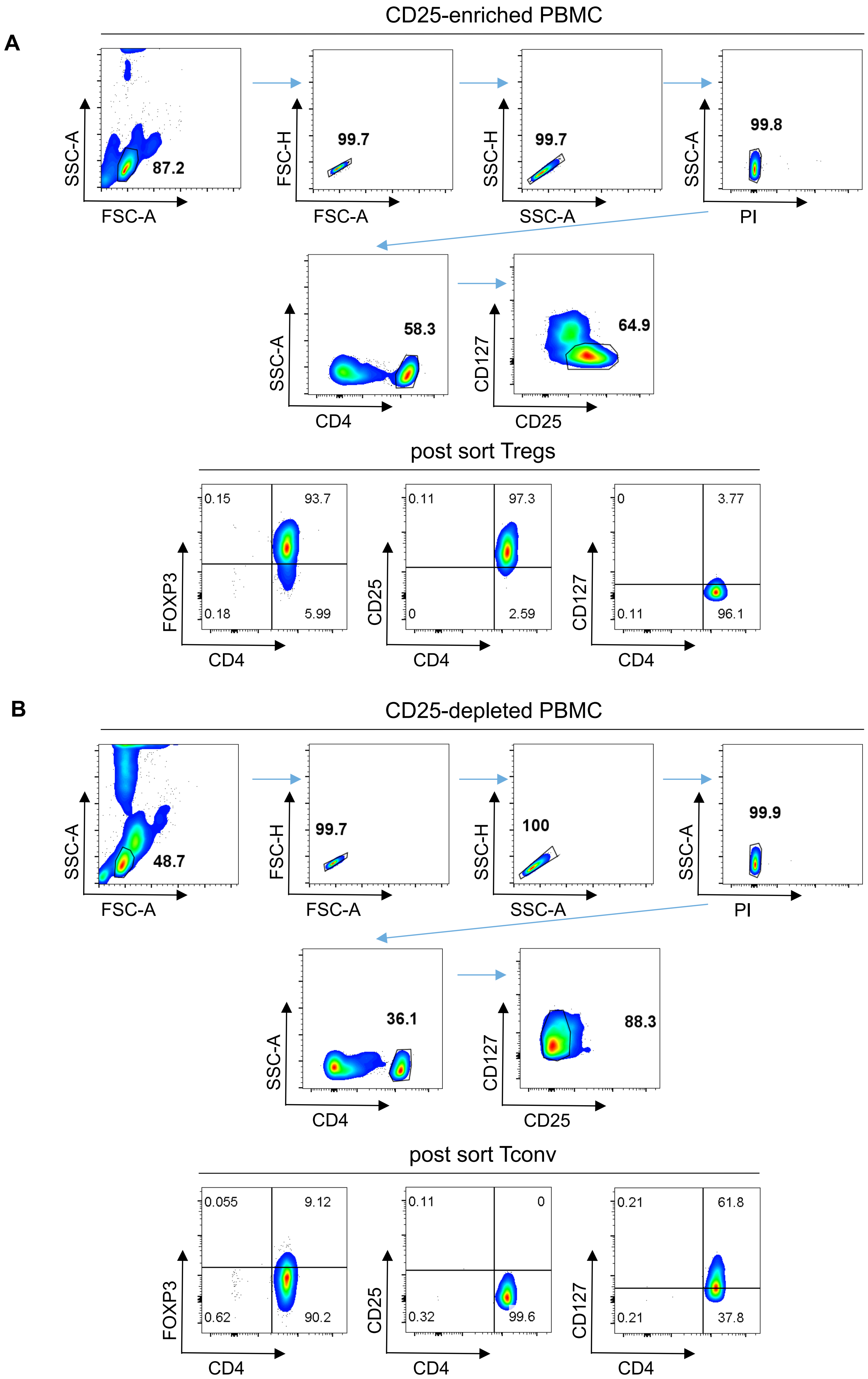

A
CD25-enriched PBMC
SSC-A
FSC-A
87.2
FSC-H
FSC-A
99.7
SSC-H
SSC-A
99.7
SSC-A
PI
99.8
SSC-A
CD4
58.3
CD127
CD25
64.9
post sort Tregs
FOXP3
CD4
0.15
93.7
0.18
5.99
CD25
CD4
0.11
97.3
0
2.59
CD127
CD4
0
3.77
0.11
96.1
B
CD25-depleted PBMC
SSC-A
FSC-A
48.7
FSC-H
FSC-A
99.7
SSC-H
SSC-A
100
SSC-A
PI
99.9
SSC-A
CD4
36.1
CD127
CD25
88.3
post sort Tconv
FOXP3
CD4
0.055
9.12
0.62
90.2
CD25
CD4
0.11
0
0.32
99.6
CD127
CD4
0.21
61.8
0.21
37.8

Figure S2

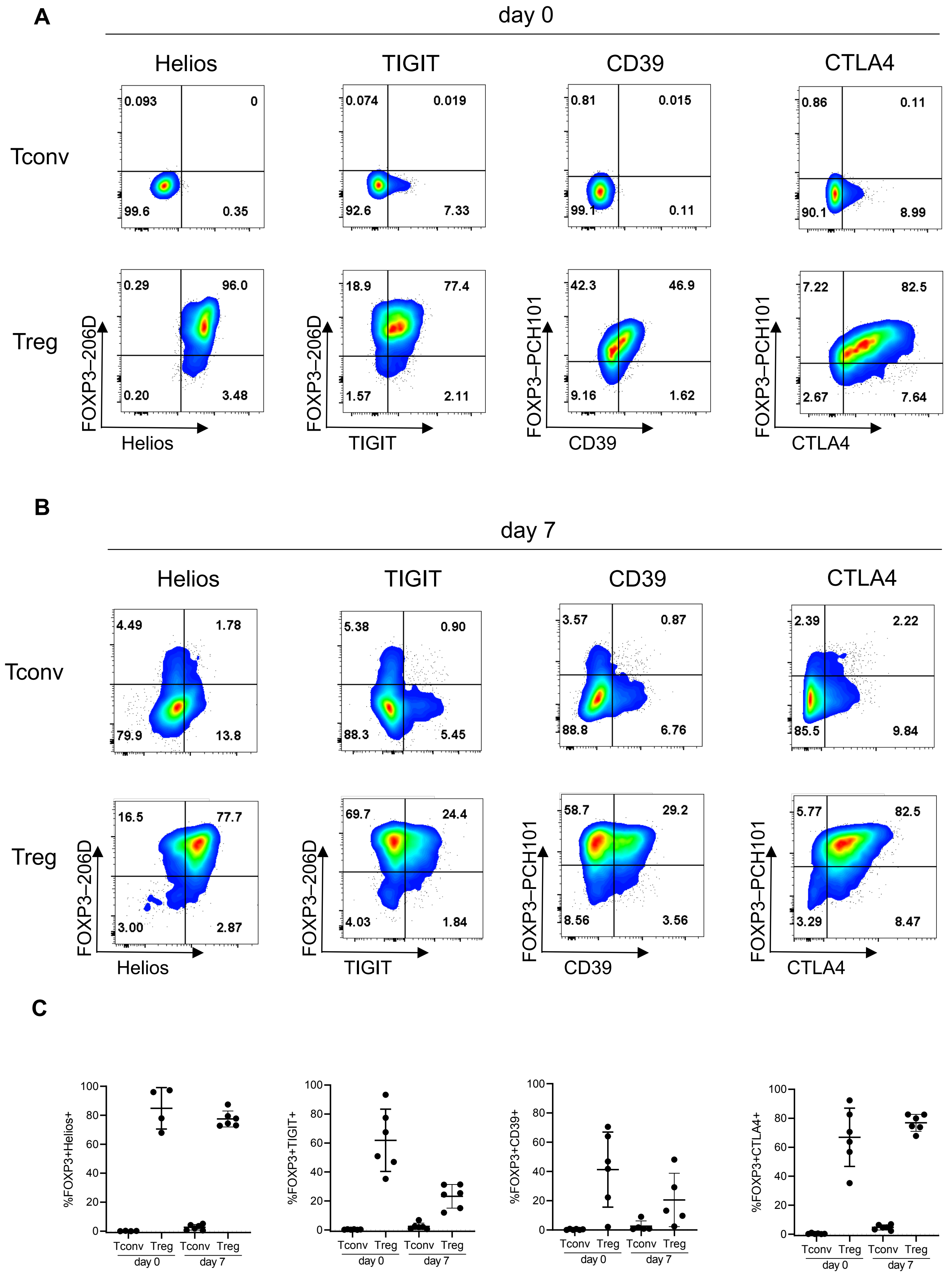

A
day 0
Helios
TIGIT
CD39
CTLA4
Tconv
Treg
0.093
0
99.6
0.35
0.074
0.019
92.6
7.33
0.81
0.015
99.1
0.11
0.86
0.11
90.1
8.99
0.29
96.0
0.20
3.48
18.9
77.4
1.57
2.11
42.3
46.9
9.16
1.62
7.22
82.5
2.67
7.64
FOXP3–206D
FOXP3–PCH101
B
day 7
4.49
1.78
79.9
13.8
5.38
0.90
88.3
5.45
3.57
0.87
88.8
6.76
2.39
2.22
85.5
9.84
16.5
77.7
3.00
2.87
69.7
24.4
4.03
1.84
58.7
29.2
8.56
3.56
5.77
82.5
3.29
8.47
C
%FOXP3+Helios+
%FOXP3+TIGIT+
%FOXP3+CD39+
%FOXP3+CTLA4+
Tconv
Treg
day 0
day 7

Figure S3

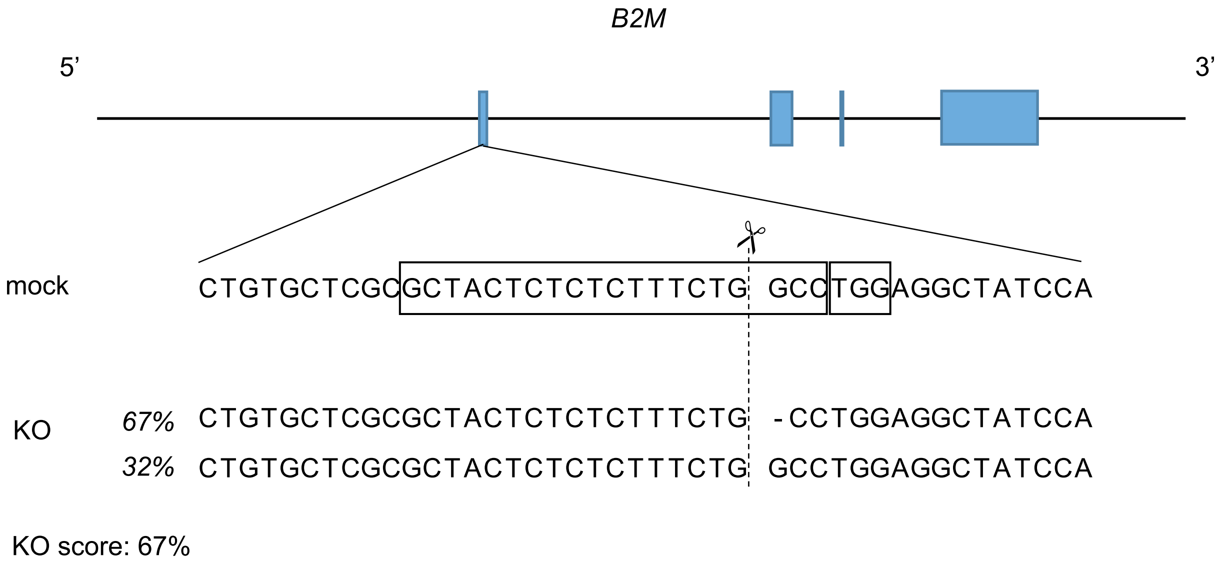

B2M
5'
3'
mock
CTGTGCTCGCGCTACTCTCTCTTTCTG GCCTGGAGGCTATCCA
KO
67% CTGTGCTCGCGCTACTCTCTCTTTCTG -CCTGGAGGCTATCCA
32% CTGTGCTCGCGCTACTCTCTCTTTCTG GCCTGGAGGCTATCCA
KO score: 67%

Figure S4

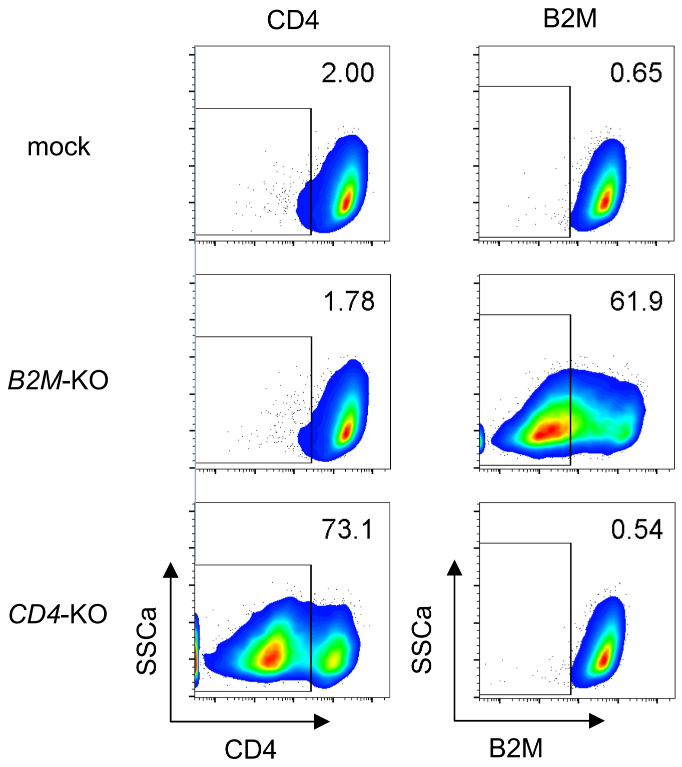

CD4
B2M
mock
2.00
0.65
B2M-KO
1.78
61.9
CD4-KO
73.1
0.54
SSCa
SSCa
CD4
B2M

Figure S5

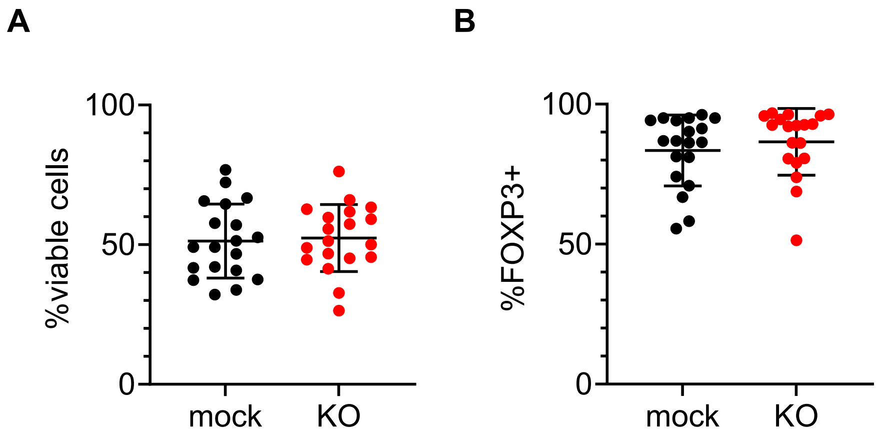

Figure S6

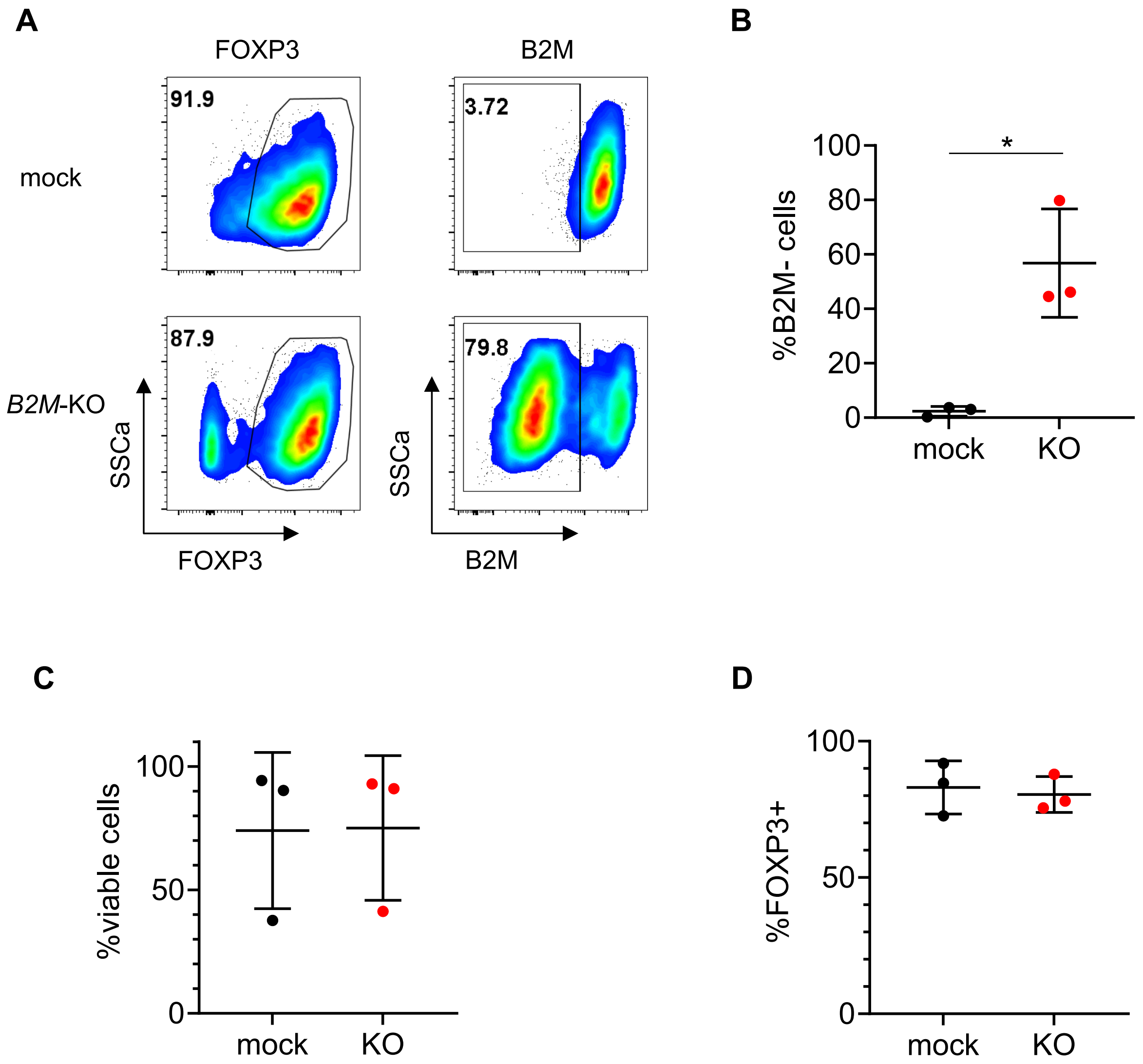
A
FOXP3
B2M
mock
91.9
3.72
B2M-KO
87.9
79.8
SSCa
FOXP3
SSCa
B2M
B
%B2M- cells
100
80
60
40
20
0
*
mock
KO
C
%viable cells
100
50
0
mock
KO
D
%FOXP3+
100
50
0
mock
KO

Figure S7

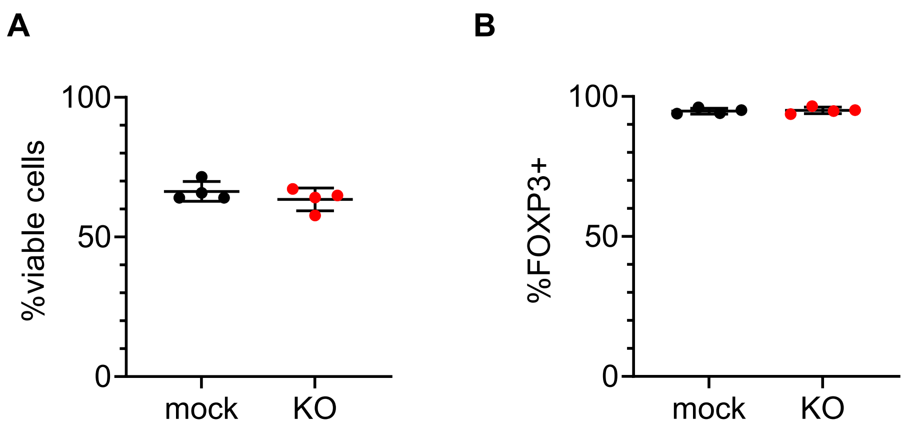

Figure S8

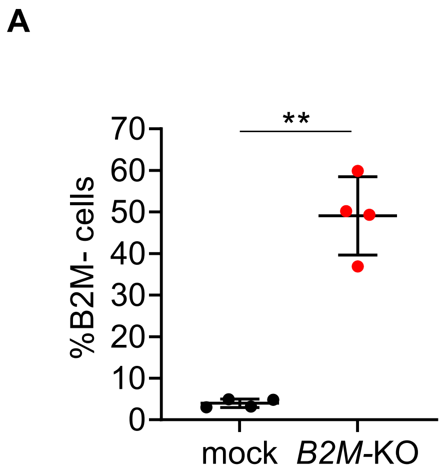
A
**
%B2M- cells
70
60
50
40
30
20
10
0
mock
B2M-KO

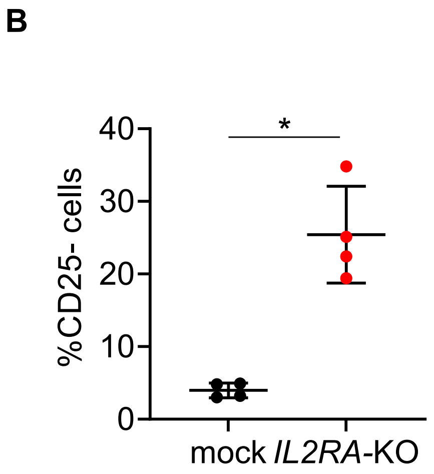
B
*
%CD25- cells
40
30
20
10
0
mock
IL2RA-KO

Figure S9

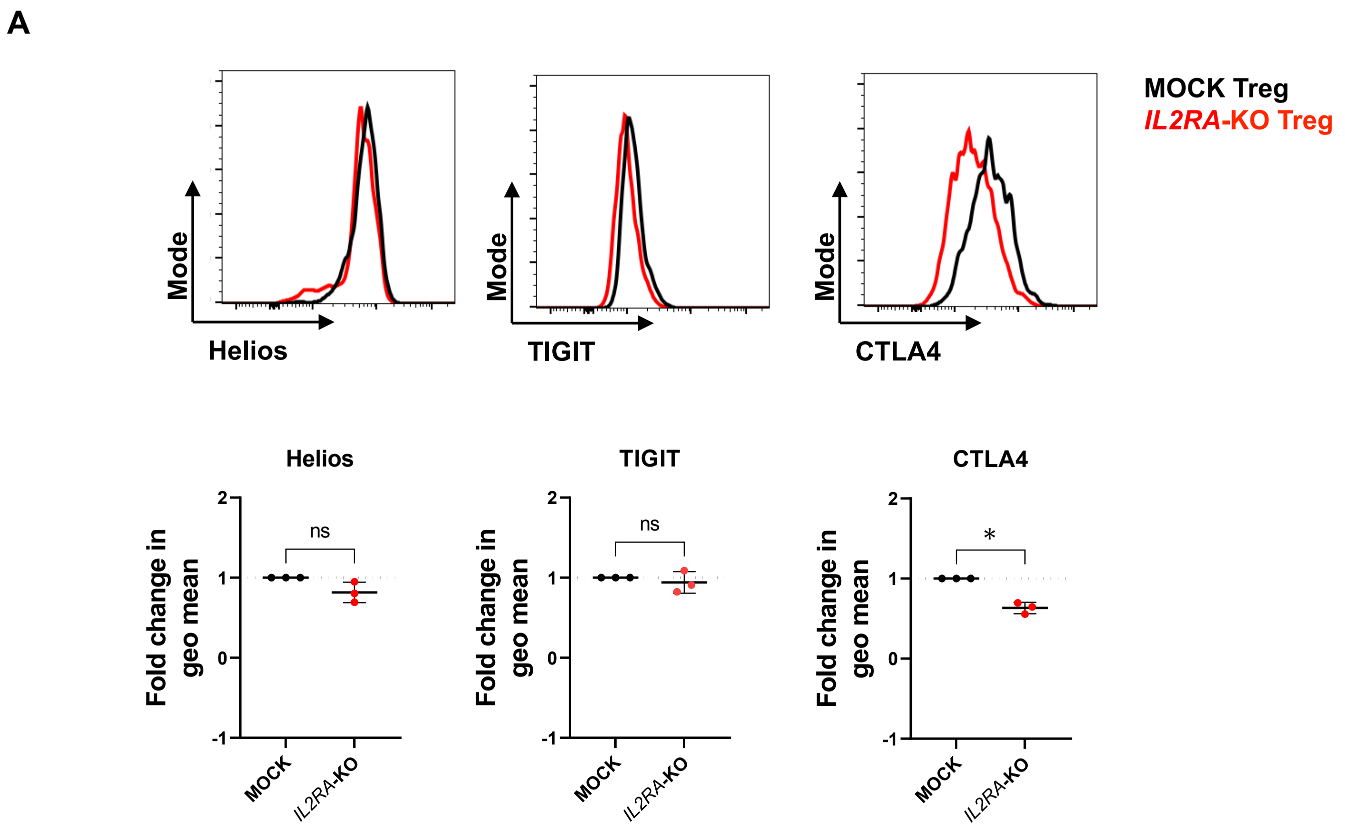
A
Mode
Helios
Mode
TIGIT
Mode
CTLA4
MOCK Treg
IL2RA-KO Treg
Helios
TIGIT
CTLA4
Fold change in geo mean
ns
ns
*
2
1
0
-1
MOCK
IL2RA-KO

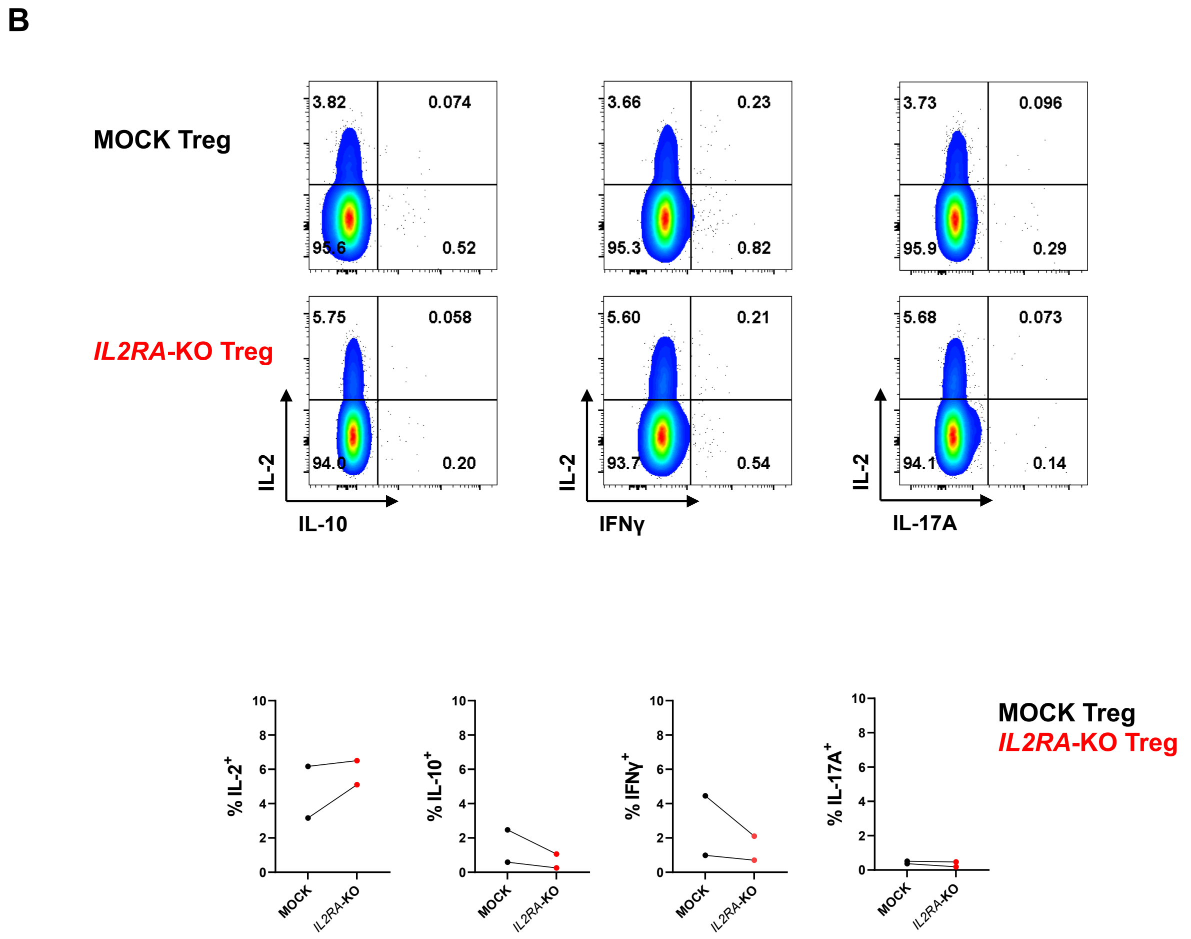
B
MOCK Treg
IL2RA-KO Treg
3.82
0.074
95.6
0.52
3.66
0.23
95.3
0.82
3.73
0.096
95.9
0.29
5.75
0.058
94.0
0.20
5.60
0.21
93.7
0.54
5.68
0.073
94.1
0.14
IL-2
IL-10
IFNγ
IL-17A
% IL-2+
% IL-10+
% IFNγ+
% IL-17A+
10
8
6
4
2
0
MOCK
IL2RA-KO
MOCK Treg
IL2RA-KO Treg

Figure S10

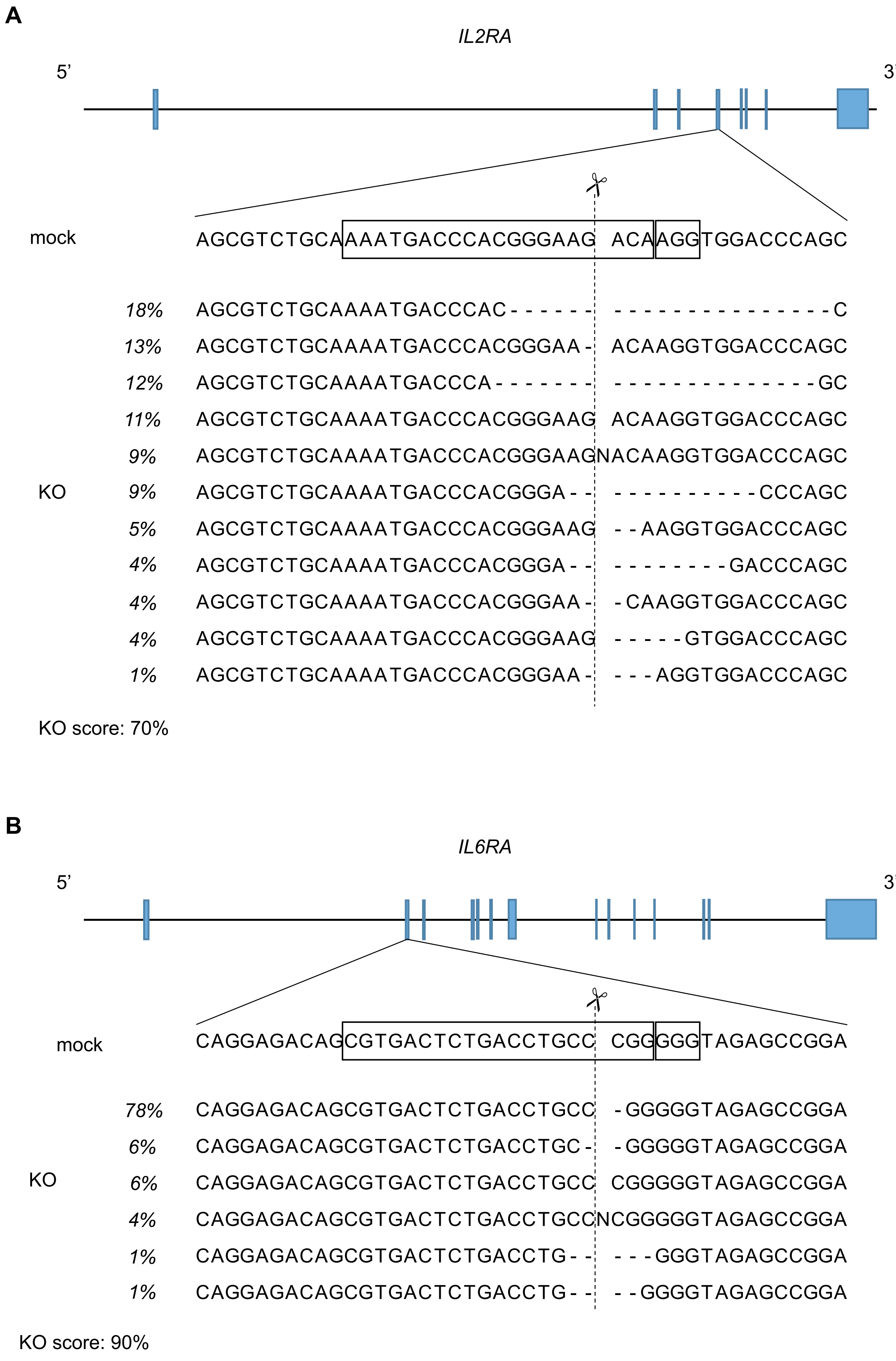
A
IL2RA
5'
3'
mock
AGCGTCTGCAAAATGACCCACGGGAAG ACAAGGTGGACCCAGC
KO
18% AGCGTCTGCAAAATGACCCAC------ ---------------C
13% AGCGTCTGCAAAATGACCCACGGGAA- ACAAGGTGGACCCAGC
12% AGCGTCTGCAAAATGACCCA------- --------------GC
11% AGCGTCTGCAAAATGACCCACGGGAAG ACAAGGTGGACCCAGC
9% AGCGTCTGCAAAATGACCCACGGGAAGNACAAGGTGGACCCAGC
9% AGCGTCTGCAAAATGACCCACGGGA-- ----------CCCAGC
5% AGCGTCTGCAAAATGACCCACGGGAAG --AAGGTGGACCCAGC
4% AGCGTCTGCAAAATGACCCACGGGA-- --------GACCCAGC
4% AGCGTCTGCAAAATGACCCACGGGAA- -CAAGGTGGACCCAGC
4% AGCGTCTGCAAAATGACCCACGGGAAG -----GTGGACCCAGC
1% AGCGTCTGCAAAATGACCCACGGGAA- ---AGGTGGACCCAGC
KO score: 70%
B
IL6RA
5'
3'
mock
CAGGAGACAGCGTGACTCTGACCTGCC CGGGGGTAGAGCCGGA
KO
78% CAGGAGACAGCGTGACTCTGACCTGCC -GGGGGTAGAGCCGGA
6% CAGGAGACAGCGTGACTCTGACCTGC- -GGGGGTAGAGCCGGA
6% CAGGAGACAGCGTGACTCTGACCTGCC CGGGGGTAGAGCCGGA
4% CAGGAGACAGCGTGACTCTGACCTGCCNCGGGGGTAGAGCCGGA
1% CAGGAGACAGCGTGACTCTGACCTG-- ---GGGTAGAGCCGGA
1% CAGGAGACAGCGTGACTCTGACCTG-- --GGGGTAGAGCCGGA
KO score: 90%

Figure S11

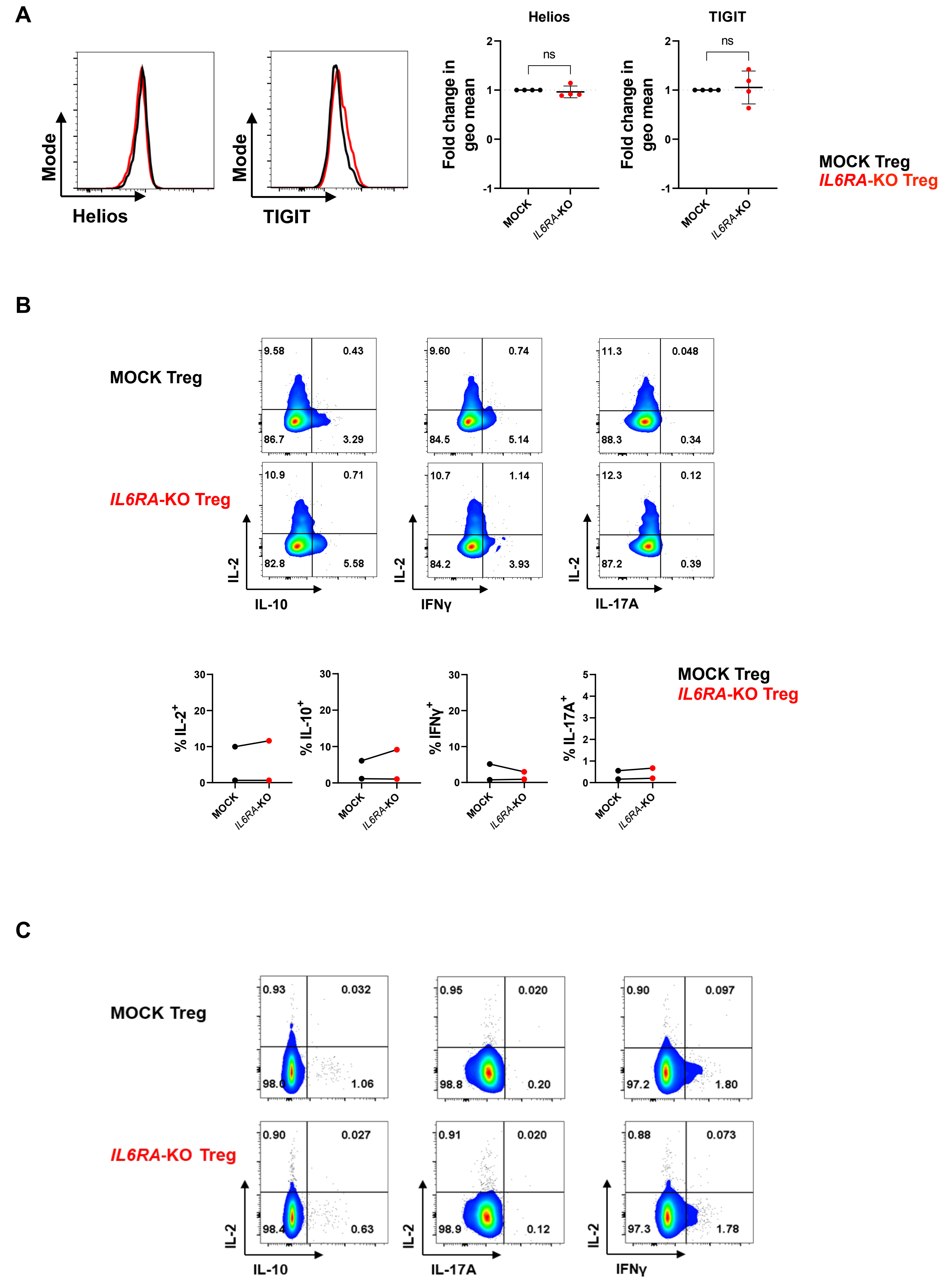

A
Mode
Helios
Mode
TIGIT
Helios
TIGIT
ns
ns
Fold change in geo mean
Fold change in geo mean
MOCK
IL6RA-KO
MOCK
IL6RA-KO
MOCK Treg
IL6RA-KO Treg
B
MOCK Treg
IL6RA-KO Treg
9.58
0.43
86.7
3.29
9.60
0.74
84.5
5.14
11.3
0.048
88.3
0.34
10.9
0.71
82.8
5.58
10.7
1.14
84.2
3.93
12.3
0.12
87.2
0.39
IL-2
IL-10
IL-2
IFNγ
IL-2
IL-17A
% IL-2+
% IL-10+
% IFNγ+
% IL-17A+
MOCK
IL6RA-KO
MOCK Treg
IL6RA-KO Treg
C
MOCK Treg
IL6RA-KO Treg
0.93
0.032
98.0
1.06
0.95
0.020
98.8
0.20
0.90
0.097
97.2
1.80
0.90
0.027
98.4
0.63
0.91
0.020
98.9
0.12
0.88
0.073
97.3
1.78
IL-2
IL-10
IL-2
IL-17A
IL-2
IFNγ
