# Supplementary material for: Fast and Efficient Genome Editing of Human FOXP3+ Regulatory T Cells
Source: Front Immunol. 2021 Aug 2;12:655122. doi: 10.3389/fimmu.2021.655122 (PMC8365355; doi:10.3389/fimmu.2021.655122)
Supplement: Supplementary file 2 [file DataSheet_1.docx]

**Supplementary tables**

*Table S1: gRNA sequences*

| **targeted gene** | **gRNA sequence + (PAM)** | **source** |
| --- | --- | --- |
| *B2M* | GCTACTCTCTCTTTCTGGCC (TGG) | Mandal et al (53) |
| *CD4* | GTCAGCGCGATCATTCAGCT (TGG) | Dang et al (74) |
| *IL2RA* | AAATGACCCACGGGAAGACA (AGG) | Roth et al (46) |
| *IL6RA* | CGTGACTCTGACCTGCCCGG (GGG) | *in-house* |

*Table S2: PCR primer sequences*

| **targeted gene** | **forward primer** | **reverse primer** |
| --- | --- | --- |
| *B2M* | GCCTTAATGTGCCTCCAGCCTG | CGACGCCCTAAACTTTGTCCCG |
| *CD4* | agctactgtcccagccaggtaaatg | agcatccctcacctgatcaagaagg |
| *IL2RA* | AGGGTCTTCGTGCCTTCCTACAG | GGACTTAGGACCAACTACGAGGCAG |
| *IL6RA* | agagatgaggcctccaaggacag | attcttgcgccaaggaaatggtgg |
